# Attention and predictions: control of spatial attention beyond the endogenous-exogenous dichotomy

**DOI:** 10.3389/fnhum.2013.00685

**Published:** 2013-10-21

**Authors:** Emiliano Macaluso, Fabrizio Doricchi

**Affiliations:** ^1^Neuroimaging Laboratory, Fondazione Santa Lucia, Istituto di Ricovero e Cura a Carattere Scientifico (IRCCS)Rome, Italy; ^2^Dipartimento di Psicologia, Università degli Studi “La Sapienza”Rome, Italy

**Keywords:** endogenous, exogenous, salience, prediction, parietal cortex

## Abstract

The mechanisms of attention control have been extensively studied with a variety of methodologies in animals and in humans. Human studies using non-invasive imaging techniques highlighted a remarkable difference between the pattern of responses in dorsal fronto-parietal regions vs. ventral fronto-parietal (vFP) regions, primarily lateralized to the right hemisphere. Initially, this distinction at the neuro-physiological level has been related to the distinction between cognitive processes associated with strategic/endogenous vs. stimulus-driven/exogenous of attention control. Nonetheless, quite soon it has become evident that, in almost any situation, attention control entails a complex combination of factors related to both the current sensory input and endogenous aspects associated with the experimental context. Here, we review several of these aspects first discussing the joint contribution of endogenous and stimulus-driven factors during spatial orienting in complex environments and, then, turning to the role of expectations and predictions in spatial re-orienting. We emphasize that strategic factors play a pivotal role for the activation of the ventral system during stimulus-driven control, and that the dorsal system makes use of stimulus-driven signals for top-down control. We conclude that both the dorsal and the vFP networks integrate endogenous and exogenous signals during spatial attention control and that future investigations should manipulate both these factors concurrently, so as to reveal to full extent of these interactions.

## Introduction

The ability to suitably allocate processing resources is fundamental for the efficient processing of incoming sensory signals and for the generation of appropriate behavior in complex environments. The brain continuously receives a large amount of sensory signals that cannot be fully processed. Attention selection allows preferential processing of some signals and filtering out of other inputs. What are the constraints that govern this selection process, thus determining what signals should gain access to in-depth processing?

Traditionally, attention research has distinguished between two types of mechanisms that contribute to the selection process. On one hand there are endogenous, top-down factors that primarily relate to the subject’s “internal” goals and intentions. On the other hand, there are exogenous, bottom-up effects that primarily relate to the characteristics “external” stimuli. Endogenous factors are associated with top-down voluntary control, while exogenous factors are associated with stimulus-driven control that is thought to take place automatically. The distinction between these two types of control can be rather intuitive: when searching for a friend in a crowd, we will voluntary shift attention from one face to another until the external input (i.e., what we see) matches our internal knowledge about the physical appearance of our friend. By contrast, in a crowd of people all dressed with dark cloths, we will quickly notice a person dressed in bright red, who will catch attention automatically. Experimentally, the impact of endogenous and exogenous factors for selection have been studied with a variety of paradigms. Somewhat related to the “real-life” examples above, visual search tasks can be used to highlight endogenous vs. exogenous factors for the allocation of spatial attention. Search tasks including a target-item “similar” to the distracters (e.g., look for a vertical red bar presented among vertical green bars and tilted red bars) will be slow and inefficient. The search time will depend on the number of competing distracters thus indicating that search proceeds serially with attention shifted voluntarily from one item to another item. By contrast, when the target item stands out (e.g., look for a red bar presented among green bars) the search is rapid and efficient, with the red target capturing attention in an automatic manner irrespective of the number of distracters.

Another way to investigate endogenous and exogenous control of spatial attention includes using variants of the Posner spatial cueing paradigm (Posner, 1980). Rather than presenting distracters during the search of a target, the target is presented in isolation but it is preceded by a spatial cue. In the endogenous version of the task, the cue is presented centrally (e.g., a leftward or rightward pointing arrow) and it predicts the target location on most of the trials (e.g., 75–80% “valid” trials). In the remaining trials the cue is “invalid” (25–20%) and the target is presented at a different position compared to the location initially signaled by the cue. Behaviorally, targets proceeded by a valid cue are detected/discriminated more rapidly/accurately than targets proceeded by invalid cues. The reason for this is that subjects can strategically use the central cue to voluntarily shift attention towards the cued location. This leads to enhanced processing when the target is presented there (valid trials), while additional re-orienting operations are needed when the target is presented somewhere else (invalid trials). In the exogenous version of this paradigm, the cue is presented peripherically (e.g., a box is flashed in the left or right visual hemifield), again followed by a target either at the same (valid) or different (invalid) location. Unlike the endogenous version, now the cue does not predict the target location (i.e., 1:1 ratio of valid and invalid trials) and the subject has no strategic reason to use the cue to voluntarily direct attention. Nonetheless, the detection of the target is faster and more accurate at the cued/valid location compared with the opposite/invalid location, provided that the interval between cue and target is short (e.g., Klein, 2000). In this case, the interpretation is that the sudden appearance of the box on one side attracts attention automatically, triggering exogenous mechanisms spatial orienting.

Many functional imaging and Event Related Potentials (ERPs) studies have investigated the neural basis of endogenous and exogenous visuo-spatial attention control. These highlighted the central role of the frontal and parietal lobes, with a notable distinction between the response patterns in dorsal and ventral regions. A variety of tasks requiring endogenous control of spatial attention highlighted the activation of dorsal fronto-parietal regions (e.g., Corbetta et al., 1993; Gitelman et al., 1999; Yantis et al., 2002). The activation pattern typically includes the intra-parietal sulcus (IPS) and/or the posterior parietal cortex (PPC) plus the frontal eye-field (FEF) in the premotor cortex. These areas are found in serial search tasks (Gitelman et al., 2002; Himmelbach et al., 2006; Fairhall et al., 2009), plus many other non-spatial attention tasks also requiring the voluntary allocation of processing resources (e.g., see Wojciulik and Kanwisher, 1999). Accordingly, the “dorsal attention network” is commonly accepted to be involved in the endogenous control of attention (Corbetta and Shulman, 2002). Furthermore, specific experimental designs allowed segregating brain activity associated with the different phases of attention tasks, which often comprise multiple control processes. In spatial cueing tasks, the dorsal attention network has been found to activate upon the presentation of the predictive cue (e.g., Hopfinger et al., 2000; Doricchi et al., 2010) and to show sustained activity in the cue-to-target delay (Kastner et al., 1999, see also Hahn et al., 2006). These preparatory effects, and the sustained activity in the absence of any external stimulus, are consistent with the “internal”/endogenous nature of the responses in the dorsal attention system. Analogous preparatory effects have been also found within the occipital visual areas that represent the attended/cued location (e.g., see also Chawla et al., 1999, Kastner et al., 1999, for related effects in non-spatial selective attention tasks). These findings support the proposal that attention selection entails top-down signals that originate in the dorsal system and modulate activity in sensory areas that represent the currently relevant location (e.g., Simpson et al., 2011). This, in turns, would yield to greater responses when visual stimuli appear at the attended location, particularly so when the display also contains other distracting visual stimuli (Desimone and Duncan, 1995; Kastner et al., 1999; Geng et al., 2006; see also Moore, 2006, for review).

On the other hand, areas in the ventral parietal and frontal cortex activate when a behaviorally relevant stimulus is presented outside the current focus of attention, for example a visual target that follows and invalid predictive cue in spatial cueing paradigms (Arrington et al., 2000). These targets stimuli are thought to capture attention in a stimulus-driven manner and the “ventral attention network” has been associated with the control of exogenous attention (Arrington et al., 2000; Corbetta and Shulman, 2002; Macaluso et al., 2002; Corbetta et al., 2008, for review). This network comprises the temporo-parietal junction (TPJ) and the inferior frontal gyrus (IFG) and is considered to be typically lateralized in the right hemisphere (Shulman et al., 2010). Nonetheless, quite soon, it has become evident that the conditions typically associated with activation of the ventral fronto-parietal (vFP) entail not only spatial aspects of attention control (i.e., the visual target triggering a shift of attention from one position to another), but they also produce some breach of expectation (e.g., the subject expects the target at one location, but this appears at a different position) and/or require some task-related judgment of the stimulus at a previously unattended location.

In the following sections we discuss recent evidence that, on one hand, highlighted the role of bottom-up signals for attention control in the dorsal fronto-parietal system and, on the other hand, demonstrated that the ventral system combines stimulus-driven aspects of spatial reorienting with endogenous signals related to expectation and the representation of task-related contextual information (predictions). These data challenge the traditional dichotomy linking the dorsal system with endogenous attention and the ventral system with exogenous attention, and indicate a novel perspective to understand the multifaceted mechanisms underlying the control of spatial attention.

## Stimulus-driven effects in the dorsal attention system

As noted above, a wealth of imaging evidence has associated activation of the dorsal fronto-parietal network with endogenous/voluntary attention control (Wojciulik and Kanwisher, 1999; Corbetta and Shulman, 2002). Nonetheless, sensory aspects of the incoming signals may also play an important role, particularly so in posterior parietal regions. Indeed, while showing increased activation even in the absence of any sensory input (Kastner et al., 1999; Hopfinger et al., 2000), the parietal cortex shows some residual spatially-specific responses also to unattended stimuli (Saygin and Sereno, 2008). The co-occurrence of sensory-driven responses and top-down internally-controlled activity, also associated with motor planning, has triggered an intense debate about the format of the spatial representations in parietal cortex (e.g., Andersen et al., 1993; Colby and Goldberg, 1999). Among the many hypotheses, the concept of “saliency map” has recently gained much interest. Saliency maps are topographical representations of the visual space and code for the relative relevance of different locations. Depending on definition, these account for bottom-up interactions between multiple sensory input/features (Itti and Koch, 2001, see also Borji and Itti, 2013) or integrate bottom-up effects with top-down goals (Gottlieb et al., 1998; Treue, 2003, for review; and Gottlieb, 2007; see also “priority maps”, Ptak, 2012). Saliency maps have been associated with neuronal responses in dorsal parietal (Gottlieb et al., 1998; Kusunoki et al., 2000; Constantinidis and Steinmetz, 2001) and dorsal pre-motor regions (Thompson et al., 2005), as well as visual areas in occipital cortex (Mazer and Gallant, 2003). In most of these regions, the patterns of response reflect both bottom-up as well as top-down factors that jointly contribute to make a region of space relevant.

In a series of imaging studies we made use of saliency maps to investigate attentional selection using naturalistic stimuli (i.e., complex pictures and videos). In order to explicitly characterize the effect of bottom-up signals, we considered the saliency model proposed by Itti and Koch (2001). This decomposes complex images into a set of conspicuity maps, representing local contrast in intensity, orientation and color, plus motion and flicker for dynamic visual stimuli (i.e., videos). These maps are then combined in a single map that represents the bottom-up salience of each location in the image, irrespective of the feature dimension making the location salient. The final saliency map reflects only sensory-driven aspects (i.e., competition between locations in the image) and can predict fixation patterns during the viewing of complex visual stimuli (Carmi and Itti, 2006; Elazary and Itti, 2008). Nonetheless, eye-movement studies have highlighted that factors other than pure bottom-up signals influence spatial orienting in complex environments (Navalpakkam and Itti, 2005; Einhauser et al., 2007; see also Borji and Itti, 2013). Accordingly, in our studies we combined saliency-related bottom-up indexes with measures derived from overt eye-movements recorded during free viewing of the stimuli. With this we sought to track the influence of bottom-up and top-down factors, and the interaction between the two, during selective processing of complex and naturalistic stimuli (see also Seidl et al., 2012).

In a first study, we investigated the impact of visual salience on spatial orienting using a virtual visual environment (Nardo et al., 2011). We computed the mean salience of each visual frame and used this as a covariate for the analysis of the imaging data. This revealed that activity in the occipital visual cortex and the PPC increased with increasing salience of the visual input (see also Bordier et al., 2013, who separated the effect of salience vs. single visual features). Notably, when we combined saliency data and patterns of eye-movements we found that activity in the entire dorsal fronto-parietal network (i.e., including both parietal and frontal nodes) increased specifically when the salient bottom-up signals successfully/efficaciously attracted the subjects’ gaze. These activations were found also when we asked subjects to maintain central fixation (i.e., now indexing the efficacy of the bottom-up signals in one session, and using these indexes to analyze the fMRI data in a different session), consistent with an interpretation based on spatial attention/selection rather than mere oculomotor control. Related results were also obtained by Bogler et al. (2011), who used multivariate pattern recognition to reveal that PPC represents “selected” saliency signals following a winner-takes-all mechanism.

Previous studies considered the role of the dorsal fronto-parietal cortex for the processing of bottom-up signals within two main frameworks. On one hand, it has been proposed that activation of the dorsal system “follows” the detection of relevant stimuli by the ventral attention system (Geng and Mangun, 2011; Vossel et al., 2012). Accordingly to this view the dorsal system generates top-down signals that modulate processing in visual cortex, which require re-setting when a relevant/infrequent “bottom-up” stimulus is presented outside the current focus of attention (Corbetta and Shulman, 2002; Shulman et al., 2009). A different prospective entails a more “direct” activation of the dorsal system due to forward input from the visual cortex. In this context, differences between the involvement of the posterior IPS and the anterior FEF nodes of the dorsal fronto-parietal system have been proposed. For example, using concurrent Transcranial Magnetic Stimulation-fMRI over either IPS or FEF, Ruff et al. (2008) showed that the inter-regional influence of FEF over visual cortex does not depend on the visual input, while the influence of IPS can change according to the current visual context. IPS-TMS affected activity in retinotopic visual areas (V1-V4) only in the absence of visual input, while activity in area V5/MT+ was modulated only in the presence of visual stimuli. The authors suggested that the difference between the effect of TMS over IPS and FEF reflects the primarily top-down nature of the FEF-to-occipital connectivity vs. the presence of strong bottom-up driving projections from visual-to-IPS areas (see also Buschman and Miller, 2007; who suggested fast bottom-up attentional signals in IPS vs. slow top-down effects in prefrontal areas). Our data with complex stimuli also showed a greater influence of the bottom-up signals in IPS with that of FEF: while both regions were modulated according to the efficacy of the salient visual input in attracting subject’s attention, the activity in IPS also co-varied with the overall stimulus salience irrespective of efficacy (cf. above and see Geng and Mangun, 2009; who reported that the saliency of non-target visual stimuli is represented in IPS but not in FEF). Moreover, our study did not show any effect of sensory salience in vFP areas that instead activated when the display included attention-grabbing events (i.e., virtual human-like characters that entered the scene at unpredictable times, Nardo et al., 2011). Overall, these results are consistent with a “direct”—rather than via ventral areas—influence of bottom-up signals in the IPS, and fit with the hypothesis that the dorsal fronto-parietal cortex codes for the current attention priorities (Gottlieb, 2007).

These findings demonstrate that in complex and naturalistic experimental situations that involve high levels of sensory competition, the dorsal fronto-parietal system responds to bottom-up salient signals. However, these responses do not only reflect the impact of the external input but also they appear to index the efficacy of these signals for driving spatial selection. Eye-movement data provide us with one index of such selection processes, but do not gives us any hint about the possible consequences of the spatial selection bias. Using a working memory (WM) task, we sought to establish whether stimulus-driven attention to salient elements of a complex image has any consequence on subsequent memories of that image (Santangelo and Macaluso, 2013). Specifically, we asked whether salient objects associated with activation of the dorsal attention network are more likely to be remembered than non-salient objects or salient objects that do not activate the dorsal system (i.e., non-efficacious salient stimuli, cf. also Nardo et al., 2011). For this, we presented pictures of complex natural scenes and—following a short delay—we probed memory for one object. Critically, the probe was extracted either from an highly salient location of the image or a location of minimal saliency (see also Fine and Minnery, 2009). Behaviorally, we found better WM performance for high than low saliency probes. Analysis of the imaging data during encoding revealed increased activation in the posterior parietal cortex when the trial included a salient probe that—later, at retrieval—would be successfully remembered. This demonstrates that the selection of salient elements in a complex environment do not only trigger on-line spatial orienting (cf. eye-movements), but also leads to in-depth processing including stimulus’ storage in WM.

The finding of an interplay between attention and WM is not surprising, given the wealth of data showing analogous patterns of activation during attention and WM tasks (LaBar et al., 1999; Corbetta et al., 2002; Anderson et al., 2010). Several accounts have been put forward to explain the role of attention in WM. One of the main proposal is that attention contributes to the maintenance of items/objects in WM and that activation of the dorsal attention system—and IPS in particular—reflects the process of shifting endogenous attention between these items (see Awh and Jonides, 2001; Majerus et al., 2007; Magen et al., 2009; and Nee et al., 2013; albeit note that the latter meta-analysis pointed to inferior rather than superior parietal areas for attention shifting in WM). Related more directly to the issue of the contribution of bottom-up signals, Bundesen et al. (2011) proposed a computational framework that integrates mechanisms of selective attention and the access to WM storage by a subset of items in multi-items displays. The model emphasizes the dynamic remapping/changes of neurons’ receptive fields and the modulation of inter-regional communication (i.e., what information is sent from lower to higher levels of the visual hierarchy), via attentional weights generated by “priority” maps (cf., Gottlieb, 2007; Ptak, 2012).

These accounts are primarily concerned with the interplay between attention and WM considering situations were multiple, unrelated items compete for the access to a limited capacity WM buffer (typically holding 3–4 items, see Todd and Marois, 2004; Xu and Chun, 2006). However, in our experiment with naturalistic stimuli (Santangelo and Macaluso, 2013) the various items/objects in the scenes were not unrelated, rather they existed in relation to each other (e.g., glasses are typically found on a table, while shoes are on the floor). This, together with the finding of an increased functional coupling between the parietal cortex and the hippocampus when subjects encoded objects that later would be correctly retrieved, lead us to interpret our results in a slightly different framework. Specifically, we suggested that prior knowledge about the global scene configuration plays a role during the encoding of the position of objects within natural scenes. This would fit with theories proposing that WM entails the activation of long-term memory (LTM) representations, and that selective attention provides a possible mechanism to activate relevant portions of the LTM system (Cowan, 1993; Oberauer, 2002). In this context, attention does not only provide “pointers” to objects that are relevant, but may also help integrating bottom-up signals with information stored in LTM.

In sum, by making use of naturalistic stimuli that entail high levels of competition for processing resources, we showed activation in posterior parietal regions when salient visual signals trigger orienting of spatial attention (Nardo et al., 2011) and when salient visual objects are successfully encoded in WM (Santangelo and Macaluso, 2013). These results indicate that bottom-up salience contributes to making a given location/object relevant (stimulus-driven selection), which in turns increases the likelihood of on-line spatial orienting towards that location and the storage in memory of information concerning that location.

Based on these findings, it would be also important to assess whether any effect of bottom-up salience is maintained or disrupted in patients with unilateral attentional deficits, as left spatial neglect (see also Section Hemispheric Lateralization and the Spatial Neglect Syndrome, below). The dorsal attention system is usually structurally intact in these patients: nonetheless, the activity of dorsal areas can be functionally reduced due to structural lesions of adjacent areas in the ventral attention system. Consistent with this, the study of visual ERPs suggested that in neglect patients bottom-up visual processing can be impaired at around 130 ms post-stimulus, at the level of structurally intact dorsal attention areas (i.e., the IPS plus dorsal occipital cortex, cf. N1 component; DiRusso et al., 2008). However, the population of neglect patients is characterized by a high between-patients variability, linked to the location and extent of brain damage, so that a corresponding variability in the processing of bottom-up salience can be expected.

## Spatial reorienting and expectancy components in the ventral attention system

As noted in the introduction, the classical comparison highlighting the activation of the vFP system involves contrasting “invalid” versus “valid” trials within spatial cueing paradigms including central predictive cues. A main assumption of this approach is that the predictive cues generate a “validity context” associated to the ratio of valid over invalid cues. The statistical contingency between cues and targets cumulates during task performance and it is thought to modify behavioral performance and brain activity related to the different operations implicated in attentional orienting. Under this assumption, one may expect that symbolic cues indicating the position of the upcoming targets at chance-level will be ineffective in biasing attention and will not produce any validity effect. Here we review evidence demonstrating that, unexpectedly and contrary to the initial assumption, effects of cue validity can be also observed within a neutral “validity context”, i.e., when valid trials are as frequent as invalid ones and cues are statistically non-predictive. These experimental conditions are of particular relevance because they dissociate the effects of spatial re-orienting on invalid trials from any violation of expectations that characterize invalid trials presented within a predictive “validity context”.

The first study that emphasized the importance of isolating the neural correlates of the violation of expectancy carried by targets presented at unexpected spatial or temporal locations is a PET investigation by Nobre et al. (1999). Through the reanalysis of data from a previous study (Coull and Nobre, 1998), these authors compared brain activations recorded during phases of a Posner-like task in which the percentage of valid trials was 100% to those recorded in phases with 60% of valid and 40% of invalid trials. Invalid trials selectively engaged inferior parietal areas bilaterally, though with a larger activation in the right hemisphere, the right lateral premotor cortex and the inferior-orbitofrontal cortex bilaterally. The authors concluded that the response of the inferior-orbitofrontal cortex to invalid trials is recruited by violation in the expected cue-target congruency and that the parietal and premotor areas might instead be prevalently involved in more exquisitely spatial-attentional processes, as the shift of the attentional focus. Although the experimental design did not include any comparison of brain activations related to blocks with frequent vs infrequent invalid trials, i.e., trials implying identical spatial-attentional operations though carrying different degrees of unexpected violation of cue-target congruency, the points made by these authors were the first that set the terms of the spatial/expectancy components in reorienting.

A few years later, Giessing et al. (2004) reasoned that although event-related fMRI designs are more suitable to study transient neural processes related to infrequent events like invalid trials in a conventional endogenous Posner task, they can be insensitive to more sustained neural processes related to reorienting of attention. To isolate both transient and sustained neural components of reorienting, these authors used a hybrid event-related/block design arranging invalid trials within blocks having different ratios of valid vs. invalid trials: 100% valid–0% invalid, 75% valid–25% invalid and 50% valid–50% invalid. This allowed comparing both transient event-related (invalid vs valid contrast independently of block type) and sustained (100% Valid–0% Invalid blocks vs. 50% valid–50% invalid blocks) brain activity related to reorienting. The study revealed common event- and block-related activations in the right intraparietal sulcus. Moreover, in the blocked design the activation of this area was positively correlated with the number of invalid trials in a block. The blocked design also revealed the activation of the right occipital-parietal junction whereas the event-related analysis isolated additional activations in the right superior parietal lobule and in the left intraparietal sulcus. Unfortunately, this study did not provide us with detailed results of the contrast between the 75% and 50% block validity conditions that would have allowed for a first glance to the spatial and expectancy components of reorienting. At a behavioral level, this study reported the maintenance of the validity effect in blocks with non-predictive cues (50% valid–50% invalid): however, one should consider that this finding can still be accounted by the general positive “validity context” of the task, since across the different types of validity blocks the majority of trials were valid (73.4%).

Capitalizing on behavioral evidence showing that the ratio of valid vs. invalid cues modulates the validity effect (Jonides, 1980, 1983; Eriksen and Yeh, 1985; Madden, 1992), Vossel et al. (2006) were the first to directly assess the influence of the validity context on neural activity related to attentional reorienting. These authors used an event-related design where, in each trial, differently colored central arrow-cues predicted with different validity the position of ensuing targets, e.g., green arrow 90% of validity vs. blue arrow 60% of validity. The comparison between trial-related brain activities (i.e., with no separation of cue- and target-related brain response) in the two validity conditions showed that infrequent invalid trials (i.e., 90% validity) determined greater activation in the right IFG and middle frontal gyrus (MFG) and in the right inferior parietal cortex (angular and supramarginal gyrus, plus the intraparietal sulcus). As in previous studies, the reaction times (RT) advantage for validly cued targets was larger in the high-validity condition (90%). The authors’ advanced alternative hypotheses for the heightened response in parietal and frontal areas observed for invalid trials presented in the high-validity context (90%). For the frontal areas, they proposed that infrequent invalid trials might be more unexpected and novel or might induce stronger prefrontal inhibition of motor responses until accomplishment of spatial reorienting. The parietal response to the validity context received two possible interpretations: (1) more demanding reorienting effort to infrequent invalid targets; (2) less frequent and thus more surprising violation of expectancy with infrequent invalid targets.

More recently, Shulman et al. (2009) investigated spatial vs. expectancy components of reorienting using a modified version of the shift/stay paradigm devised by Yantis et al. (2002). Participants had to detect a target-object that was presented within one of two visual streams of object-groups, one presented to the left and one to the right side of central fixation. At the beginning of each stream-trial, the stream to be attended was cued by a peripheral red box shortly appearing to the left or the right of fixation. Crucially, streams were presented within three different block-conditions: (1) in a first condition the probability that on ensuing stream-trials the red-cue shifted from one side of fixation to the other was high (86% shift cues) whereas the probability of remaining on the same side-stream was low (14% stay cues); (2) in a second condition the probability of occurrence of shift and stay cues was equal (50%); (3) in a third condition the probability of shifting attention from the left to right of fixation or viceversa was low (14% shift cues) while the probability of remaining on the same side-stream was high (86%). In this way reorienting of spatial attention to the peripheral red box was made orthogonal to the likelihood of operating reorienting. In other words, spatial reorienting and unexpectedness of reorienting were operationally dissociated among blocks with different shift/stay probabilities. Analysis of Blood oxygen-level dependent (BOLD) signal revealed that the right TPJ was significantly activated by shift cues independently of the likelihood of reorienting (note that this is at variance with previous data by Vossel et al. (2006), showing sensitivity of the right TPJ to the expectancy component of reorienting; see also below for a further discussion of this). In contrast, the response of the right IFG was strongly influenced by unexpected shifts of attention (High Stay/Low Shift cuing condition). The basal ganglia and the frontal insular region were also activated by unexpected shifts of attention: however, analysis of resting state connectivity demonstrated that this network was functionally separated from the classical TPJ-IFG ventral system. The authors proposed that the response of the basal ganglia-insular network might be linked to the change in the attentional set, or to higher inhibition of competing attentional processes elicited by unexpected peripheral cues triggering reorienting of attention. Finally, dorsal attentional areas in the IPS and FEF showed intermediate sensitivity to expectancy: according to the authors the increased response of these areas to unexpected shifts of attention was driven by expectedness-related responses in the right IFG and in the basal ganglia-insular network.

The studies summarized above investigated the influence of the “validity context” by examining brain responses related to the entire cue-target period or to peripheral stimuli that acted both as attentional relevant stimuli and cues (Shulman et al., 2009). However, one important finding in the study of attention is that cumulated knowledge about the occurrence of previous stimuli (trials’ history) can influence the focusing of attention ahead of the occurrence of new relevant stimuli. Shulman et al. (2007) showed that the more target occurrence in a rapid visual stream becomes probable along the timeline, the more TPJ is deactivated. Put in other words, the response of the attention controlling networks is modulated according to the likelihood/predictability of a given target type to occur. With respect to studies using the Posner task with central cues, the observation by Shulman et al. (2007) highlights the importance of disentangling the influence of the validity context on cue- and target-related brain activity.

With this aim we recently reinvestigated the influence of cue-predictiveness on cue- and target-related brain activity (Doricchi et al., 2010). Two groups of participants were considered: one performed an endogenous Posner task with highly predictive cues (80% valid–20% invalid trials) whereas the other performed the task using non-predictive cues (50% valid–50% invalid trials). Keeping a fixed cue-target probabilistic relationship during the entire task allowed avoiding the possible influence of strategic factors related to trial-by-trial or block-by-block changes in the probabilistic cue-target congruency. In the same study, we also introduced spatially-neutral cued trials to assess the influence of the validity context on attentional benefits (RT advantage to validly cued vs neutrally cued targets) and costs (RT advantage to neutral vs invalidly cued targets). We found that during endogenous orienting, predictive cues lead to a greater deactivation of the right TPJ as compared to non-predictive ones. Since deactivation of the TPJ has been previously related to the filtering out of unattended events (Shulman et al., 2007), this finding suggests that when cues are not predictive and invalid targets frequent, the TPJ reduces the filtering out of uncued locations to facilitate reorienting. The study of target-related activation showed that, compared to valid targets, frequent and infrequent invalid targets equally activated the right TPJ, whereas infrequent invalid targets produced a stronger response in the right IFG and MFG: this shows that the TPJ is sensitive to the simple mismatch between cued and actual target location and that IFG-MFG are sensitive to the unexpectedness of such a mismatch. Both the left and right TPJ displayed no preference for targets presented in the ipsilateral or contralateral space (unpublished data). No effect of validity context was found in cue- and target-related responses of dorsal attentional areas (IPS and FEF). This seems at variance with findings by Shulman et al. (2009): this different result, however, could be accounted on the fact that in our study the validity context was stable, whereas in the study by Shulman et al. (2009) the predictiveness of peripheral shift/stay cues was randomly alternated between 14%, 50%, and 86% across the 20 blocks of trials. Finally, unlike the right IFG/MFG that was modulated according to expectancy (cf. infrequent invalid targets), the left TPJ and left IFG responded to frequent valid targets matching cue-related expectations. In agreement with these results, DiQuattro and Geng (2011) reported activation of the left TPJ and IFG in response to salient contextual stimuli predicting, i.e., matching, the concomitant task-related stimuli.

The comparison of brain and behavioral responses to valid and invalid targets with those to neutrally cued ones provided a number of additional functional and behavioral observations. Independently of cue predictiveness, valid targets activated the left TPJ, whereas invalid targets activated both the left and right TPJs. These findings suggest that the selective activation of right TPJ that is usually found in the direct comparison between invalid and valid trials (but see below for a number of studies reporting bilateral TPJ response to invalid targets) may result from a common response to both valid and invalid target in the left TPJ. At the behavioral level, in the non-predictive condition the validity effect was reduced, though, not abolished. This is important because it shows that even when the general validity context of an endogenous Posner task is constant and neutral, non-predictive cuing can still bias participants’ attention. Even more importantly, the analyses of the benefits and costs, showed—quite surprisingly—that the reduction of the validity effect in the non-predictive condition was entirely explained by a drop in the costs; whereas benefits were equivalent in the predictive and non-predictive conditions (it is worth noting that only participants showing a reliable validity effect, i.e., RT advantage to valid vs. invalid targets, during a training pre-test session with 80% valid cuing were included in the study). All together these findings show that: (a) the left TPJ hosts both neuronal populations coding the mismatch between cued and actual target location on invalid trials and cue-target matches on valid trials; (b) the right TPJ hosts neuronal populations coding the mismatch between cued and actual target location; (c) the validity context modulates TPJ activity during the cue period but shows no comparable influence on target-related brain response: this suggests that in fMRI studies investigating the influence of the validity effect it is suitable to disentangle cue- and target-related effects. Cue- vs. target-related effects also provides us with a possible explanation for the different findings between the study of Vossel et al. (2006), which reported sensitivity of the TPJ to the validity context, and those by Shulman et al. (2009) who did not report any such effect. In the study by Vossel et al. (2006) there was no separation between central/cue- and peripheral/target-related brain responses, whereas in the study by Shulman et al. (2009) only peripheral/target-related responses were studied. In our study (Doricchi et al., 2010), cue- and target-related brain responses were investigated separately. This showed that the right TPJ is sensitive to expectancy during the cue-period, when it showed a greater deactivation for informative compared to non-informative cues, though not during the target period, when it showed equivalent levels of activity both in the low- and high-expectancy conditions. These results suggest that in Vossel et al. (2006) the expectancy effects in the right TPJ may have been partially due to cue-related activity, whereas the effects in the right IFG-MFG can be attributed to the sensitivity of these areas to target-related expectancy, i.e., greater responses to unexpected/infrequent invalid targets.

In two other studies we investigated the response pattern of the vFP system seeking to further dissociate pure stimulus-driven effects from expectation and task-relevance (Natale et al., 2009, 2010). In one study (Natale et al., 2010), we made use of an attention capture paradigm with non-predictive cues (Folk et al., 1992) to investigate whether task-irrelevant visual features associated with the target modulate activity in TPJ-IFG. The paradigm consisted in classical exogenous cueing task, with a box briefly flashed in one or the other hemifield, shortly followed by a task-relevant visual target at the same (50% valid trials) or the opposite side (50% invalid trials). By virtue of this 1:1 ratio between valid and invalid cues, the experiment entailed a fully neutral “validity context”. The target was a triangle presented within the box and the task of the subjects was to report whether the triangle was pointing upward or downward. Critically, here we manipulated the color of the targets (red or blue) and the color of the cues (red or green). Despite the red/blue color of the target was fully task-irrelevant, at the behavioral level we found larger effects of cue-validity (invalid vs. valid trials) when the cue was red (set-relevant cues: same color as the target set) than when the cue was green (set-irrelevant cues: color not included in the target set). The imaging data revealed that invalid set-relevant red-cues activated the right TPJ, irrespective of target color; while invalid trials with set-irrelevant green-cues did not activate the TPJ. These results confirm that in the absence of any relationship between cues and targets (green cue + red/blue target), exogenous invalid cues do not trigger any spatial selection processes in the ventral attention system. However, it is sufficient that the cue shares some propriety with the current task-set (here, “redness”) to activate the ventral network, even in the absence of any breach of expectation. Since this effect was specific for the invalid trials (vs. valid trials), we assumed that it arose at the onset of the target. However, because of the specific timing of the stimuli, we were unable to separately assess the effect of set-relevance on cue-related vs. target-related activity (i.e., targets were presented immediately after the cues) nor whether any differential pattern of de-activation compared to resting state activity played any role here (i.e., the range of inter-trial intervals was only 1.9–3.0 s; cf. Shulman et al., 2007). Nonetheless, these results highlighted that external signals—specifically, the spatial relationship between the cue and the target—and internal information about the current task-set jointly contribute to the re-orienting effects in ventral attention system (see also Serences et al., 2005).

Another approach that enabled us to investigate the interplay between non-predictive signals and endogenous task-settings involved using a double-cue paradigm (Berger et al., 2005). The aim of a double cue paradigm is to engage endogenous and exogenous attention control concurrently within the same trial, thus providing a direct measure of whether/how these two mechanisms interact with each other. Specifically, the presentation of a peripheral non-predictive cue after a predictive endogenous cue enables studying the effect of a fully task-irrelevant and non-predictive stimulus (i.e., the exogenous cue) presented under conditions of top-down focused attention. This may be important when considering that the typical situation yielding to activation of TPJ-IFG involves shifting attention from a relevant location (e.g., that signaled by a predictive cue) to another relevant location (i.e., the position of an invalidly cued target). On each trial we presented first an endogenous predictive cue, then an exogenous peripheral cue and, finally, the visual target (Natale et al., 2009). The results highlighted activation of the vFP network when the endogenous cue was invalid, irrespective of the validity/invalidity of the exogenous cues. This demonstrated that this network does not process task-irrelevant and non-predictive stimuli, even when attention has been endogenously focused and the task-irrelevant stimuli provide spatial information that mismatches the current spatial expectations (i.e., trials including “invalid” exogenous cues). These results support the view that activation of the ventral attention system is involved in stimulus-driven updating of spatial expectations, only when the stimulus (e.g., a task-relevant target or a set-relevant cue) signals a “new” location that is potentially relevant.

To summarize, there is now ample evidence that activity in the vFP system reflects some interplay between stimulus-driven factors (e.g., the onset of an unexpected stimulus) and other endogenous/internal constraints. Mere bottom-up stimulus onset is insufficient to activate this network (e.g., Kincade et al., 2005; Indovina and Macaluso, 2007), while the specific relationship between the characteristics of the external signal and the internal goals/expectations plays a pivotal role for the activation of this system (e.g., see Corbetta et al., 2008, for review; Natale et al., 2010). This relationship may be relatively direct, e.g., the stimulus requires some overt judgment/response despite breaching current expectations (e.g., a task-relevant invalid target, following a predictive cue); or can be more subtle, e.g., the stimulus is task-irrelevant but still taps into task constraints that are currently relevant (e.g., contingent capture paradigms).

## Integration of endogenous and exogenous signals: open issues and future work

### Hemispheric lateralization and the spatial neglect syndrome

Despite the common assumption that mechanisms of attentional reorienting are predominantly—if not exclusively—homed in the right hemisphere (Shulman et al., 2010), a number of studies have reported bilateral rather than right unilateral activation of the TPJ in response to unexpected and invalid targets (Serences et al., 2005; Asplund et al., 2010). Through comparisons with neutral trials, we have recently shown that the left TPJ activation to invalid targets is often missed because this area responds both to invalid and valid targets (Doricchi et al., 2010). Here, we wish to emphasize that these effects in the left hemisphere can help understanding the reorienting deficits in neglect patients. The observation that the same regions that in the right ventral attention network (TPJ and IFG-MFG) are activated by reorienting towards both sides of space are also the most frequently damaged in patients with left spatial neglect (Doricchi and Tomaiuolo, 2003; Mort et al., 2003; Bartolomeo et al., 2007; He et al., 2007; Doricchi et al., 2008; Verdon et al., 2010) was taken as evidence to explain the higher incidence of neglect after right brain damage (Corbetta et al., 2008). However, assuming an exclusive competence of the right ventral network in reorienting towards both side of space would imply that damage to this network should cause a bilateral reorienting deficit. At variance with this prediction, neglect patients show severely impaired reorienting towards invalid targets in the contralesional left side of space, but no comparable ipsilesional deficits (Posner et al., 1984; Morrow and Ratcliff, 1988; Friedrich et al., 1998; Losier and Klein, 2001; Vossel et al., 2010; Rengachary et al., 2011). A possible explanation of this is that the residual ipsilesional reorienting abilities in neglect patients are based on the reorienting response of the intact left hemisphere. The competence of the left ventral network in detecting both cue-target matches on valid trials and cue-target mismatches in invalid trials may also explain the preserved ability of neglect patients in representing and exploiting the statistical contingency governing the spatial distribution of attentional relevant stimuli (Bartolomeo et al., 2001; Geng and Behrmann, 2002). We advise reconsidering hemispheric lateralization during spatial re-orientating, as this will potentially refine our understanding of pathologies associated with deficits of spatial attention.

### Early or late attentional function for the TPJ?

Electrophysiological investigations suggest that the TPJ plays a crucial role in the late phases of attentional processing, when it is believed to generate the P300b component signaling the match or mismatch between actual and predicted sensory input (Knight and Scabini, 1998). Capitalizing on this evidence we have proposed (Doricchi et al., 2010) that the response of the right and left TPJ to invalid targets may reflect the activity of a late-processing “MisMatch” system and the response of the left TPJ to valid targets that of a complementary “Match” system. Both systems would provide signals that help the brain in updating the internal models of statistical cue-target congruency which, in turn, would help keeping or switching the attentional task-set and the building-up of predictions about the position of upcoming targets. DiQuattro and Geng (2011) recently have provided evidence showing that one important function of the left hemispheric “Match” system concerns the processing of salient visual contextual cues that regularly predict, i.e., match, the position of concurrent but less salient targets. Interestingly, while the reorienting of attention after invalid central cues does activate the right (and left) TPJ, reorienting after invalid exogenous peripheral cues does not (e.g., Kincade et al., 2005). Based on our interpretation of the role of TPJ, this finding suggests that a key difference between exogenous and endogenous orienting is that only in the latter case a template of the expected target can be prepared during the cue period and then compared, through a Match/MisMatch process, with the actual target.

However, in a recent fMRI study Vossel et al. (2012) provided evidence challenging the idea that spatial reorienting is first initiated in dorsal parietal areas and that the TPJ comes into play only in a late phase of target processing. These authors used dynamic causal to characterize the effective connectivity within and between the ventral and dorsal attentional system during orienting to valid targets and reorienting to invalid targets. One key finding of this study was that invalid cuing enhanced connectivity from the ventral right TPJ to the dorsal right IPS rather than viceversa. The authors concluded that the violation of the expected cue-target congruency signaled by the TPJ may precede and help the reorienting-related shift of spatial attention governed by dorsal areas.

The notion that the allocation of processing resources involves some comparisons between expectations stored in “internal models” and the incoming sensory input (cf., Match/MisMatch systems, above) bears some relationship with recent proposals concerning the role of “predictive coding” in visual processing (Rao and Ballard, 1999). These postulate a hierarchical organization of processing where higher-order nodes represent the expected signal and inform lower-level nodes about this prediction (top-down influences). Upon stimulation, the input is compared with the predictions and any resulting error is fed-forward in order to update the internal model. Such architectures have been used to explain several visual phenomena (e.g., extra-classical receptive fields, Rao and Ballard, 1999) and, more recently, expectation-related effects in visual attention (Spratling, 2008; see also Summerfield and Egner, 2009, for review; Feldman and Friston, 2010). In this context, low probability invalid trials may generate a prediction error, possibly with an additional update of an internal model that would keep track of the probabilistic relationship between the positions signaled by the cues and where the targets are actually presented. Nonetheless, we should notice that a central tenet of predictive coding concerns the hierarchical organization of processing, which appears suitable to describe attention-related effects within occipital areas (e.g., Spratling, 2012, who implemented predictive coding to model saliency-related effects in primary visual cortex) or between high-order parietal (frontal) regions and visual areas (see also den Ouden et al., 2010, for a possible role of sub-cortical structures); but may be less appropriate to explain interactions between the dorsal and the vFP networks or between the left and the right TPJ.

In summary the interplay between dorsal areas adjacent to the IPS and the ventral TPJ appears to be an important challenge for future studies. These will have to be cautiously taken into account that a given area can show both fast and slow responses to invalid targets (Chambers et al., 2004) and that the interaction between different visual-attentional areas can be characterized by complex reciprocal exchange of feedback/forward signals. In addition it would of interest to investigate the role played by the ventral and dorsal attentional network when the statistical predictiveness of cue stimuli is updated and exploited to anticipate upcoming target events. Available evidence allows us to hypothesize that the ventral attentional system may perform a dynamic, trial-by-trial evaluation of the cue-target contingency and that the results of this is fed to dorsal areas. In turn, dorsal regions would make use of this information to update higher-order salience/priority maps that—via top-down control—modulate the processing of incoming signals in occipital sensory areas. This may be tested using TMS perturbations of the TPJ and dorsal parietal regions at different intervals, following the presentation of cues with variable validity/predictiveness.

### One or multiple filters for the allocation of attentional resources?

We reviewed several studies showing that the validity context modulates the validity effect: the higher the statistical predictiveness of the cues, the higher the RT advantage for valid as compared to invalid targets. This relationship has been modeled as the result of a single-filtering operation, where the ratio between the accumulation of attentional resources at the attended position and the withdrawal of resources from unattended locations is positively correlated to the predictiveness of the cues. However, we have recently found that the reduction of the validity effect with non predictive endogenous cues (i.e., neutral “validity context”) can be selectively driven by the abatement of attentional costs, whereas benefits can be maintained with both predictive and non predictive cuing (i.e., with both positive and neutral “validity contexts”). In an ensuing ERPs study (Lasaponara et al., 2011) we have recently found that the abatement in attentional costs is matched with the disappearance of differences in the amplitude of the P1 wave evoked by invalidly vs. neutrally cued targets (see Luck et al., 1994). By contrast the maintenance of benefits in predictive and non-predictive cuing conditions is paralleled by larger N1 amplitude in response to validly vs. neutrally cued targets. This difference between the effect of predictiveness on costs and benefits cannot be easily accounted for by a single filtering mechanism. A linear relationship between the level of cue predictiveness and the ratio between the activation of the cued vs. the uncued location would predict a symmetrical reduction of benefits and costs with non-predictive cuing. A more articulated interpretation is needed (Lasaponara et al., 2011).

At a neurophysiological level, visual-spatial orienting is regulated by the combined action of different—though functionally related—pools of visual, visuomotor and saccadic neurons within the dorsal fronto-parietal network (e.g., in the intraparital cortex: Ipata et al., 2006; Thomas and Parè, 2007; Superior Colliculus: McPeek and Keller, 2002; and in the FEF: Bruce and Goldberg, 1985; Schall and Hanes, 1993; Hanes and Schall, 1996; Thompson et al., 1996, 1997; Sommer and Wurtz, 1998; Bisley and Goldberg, 2003; Juan et al., 2004; Schall, 2004; Thompson et al., 2005). This allows hypothesizing synergic filtering mechanisms, via anatomically separated but functionally related pools of neurons. Some degree of independence between these pools can provide us with an explanation for the differential effect of the “validity context” on benefits and costs. For example, in the case of non-predictive cuing, the activation of visuomotor responses directed toward the cued location could guarantee the preservation of attentional benefits, whereas concomitant visual selection of both cued and uncued locations might help reducing attentional costs. In humans, some segregation between mechanisms of visual and visuomotor selection was demonstrated in cortical areas traditionally involved in saccadic programming. Muggleton et al. (2003) showed that the inactivation of the FEF by TMS stimulation slows down the selection of poorly salient or non-predictable visual targets in visual search tasks (see Thompson et al., 1997, for equivalent neurophysiological findings in the macaque). These observations highlight the need of exploring further the complex and multi-stages mechanisms that regulate the strategic allocation of attentional resources in space.

## Conclusion

Traditional views of attention control posit a distinction between endogenous control in dorsal fronto-parietal regions and stimulus-driven control in vFP areas. However, in recent years, such a strict dichotomy has been challenged. Here we reviewed evidence that the dorsal system makes use of bottom-up salient signals to select relevant elements in complex environments; and that the processing of external stimuli in the ventral system takes into account endogenous factors associated with the experimental context (e.g., task-set, expectations, predictiveness). We emphasize that attention control must pick up statistical ir-/regularities of the environment and integrate these with on-line information about the current sensory input. This interaction determines the selection of the most relevant stimuli and governs the allocation of the attentional resources. At the physiological level, this is likely to require some interplay between the dorsal and the ventral attention systems. We propose that the ventral system performs moment-to-moment match/mismatch operations, comparing current expectations/predictions with the actual sensory input. The result of these operations leads to a continuous update of the expectations and predictions, which the dorsal system utilizes to control the allocation of spatial attention.

## Conflict of interest statement

The authors declare that the research was conducted in the absence of any commercial or financial relationships that could be construed as a potential conflict of interest.
